# Does the presence of Q waves on ECG indicate myocardial scar on cardiac MRI?

**DOI:** 10.1186/1532-429X-11-S1-P265

**Published:** 2009-01-28

**Authors:** Gurjit Singh, Leonidas Tzogias, Mario Njeim, Milan V Pantelic, Khalid Nour, Mouaz H Al-Mallah

**Affiliations:** Henry Ford Hospital, Detroit, MI USA

**Keywords:** Cardiac Magnetic Resonance, Anterior Segment, Cardiac Magnetic Resonance Imaging, Clinical Reason, Myocardial Necrosis

## Background

Pathologic Q waves are the classic ECG sign of myocardial infarction. Prior studies have shown cardiac MRI is a more sensitive tool to detect myocardial necrosis. However, it is unclear whether pathologic Q waves on ECG always correlate with ischemic pattern of delayed enhancement. We tested the hypothesis that Q waves always indicate the presence of myocardial scaring on contrast enhanced cardiac Magnetic resonance imaging.

## Methods

We included 41 (25% females) consecutive patients with pathologic Q waves on ECG who underwent contrast enhanced cardiac MRI (1.5 T) for various clinical reasons. Imaging protocols included cine SSFP sequence and post double dose contrast delayed imaging. Q waves were considered pathologic if the total Q wave voltage is more than one third of the QR voltage. Left ventricular myocardial scar was evaluated qualitatively using the AHA recommended 17 segment model by 2 readers who were blinded to the clinical and ECG data.

## Results

A total of 29/41 (69%) patients had evidence of myocardial scar in coronary pattern, 18 (44%) of which were transmural. The presence of a Q only pattern (absence of R or S waves) did not improve the accuracy of ECG in identifying myocardial delayed enhancement on MRI. However, Q waves only modestly localized the scar. Anterior Q waves (64%) have the best correlation with the presence of scar in the anterior segments with lateral and inferior being 41% and 40% respectively. In addition, the Q only pattern did not improve the localizing accuracy of ECG.

## Conclusion

The presence of pathologic Q waves on ECG is not always associated with the presence of prior myocardial infarction on cardiac MRI. In addition, Q waves appear to modestly correlate with the location of the prior MI. Further studies should evaluate the potential causes of pathologic Q waves in patients without prior myocardial infarction.

